# Molecular interactions at the interface: polyoxometalates of the Anderson-Evans type and lipid membranes

**DOI:** 10.3389/fchbi.2024.1454558

**Published:** 2024-10-11

**Authors:** Alina A. Pashkovskaya, Nadiia I. Gumerova, Annette Rompel, Elena E. Pohl

**Affiliations:** 1 Physiology and Biophysics, Department of Biological Sciences and Pathobiology, University of Veterinary Medicine, Vienna, Austria; 2 Universität Wien, Fakultät für Chemie, Institut für Biophysikalische Chemie, Wien, Austria

**Keywords:** polyoxometalates, zeta potential, liposome, polyoxotungstates, polyoxomolybdates, DOPE, potassium fluoride, Anderson-Evans structure

## Abstract

Polyoxometalates (POMs) are metal-oxygen clusters composed of {MO_6_} octahedra that have attracted considerable attention due to their remarkable antiviral, antibacterial and antitumor activities. Despite their potential, the molecular mechanisms underlying their cellular toxicity remain poorly understood. This study investigates how Anderson-Evans type polyoxotungstates (POTs) and polyoxomolybdates (POMos) interact with biological membranes by examining their effects on the zeta (ζ) – potential of the lipid bilayer and the size of small unilamellar liposomes of different phospholipid compositions. POTs affected the ζ-potential of neutral (1,2-dioleoyl-sn-glycero-3-phosphocholine, DOPC) and slightly negatively charged (1,2-dioleoyl-sn-glycero-3-phosphoethanolamine; DOPC:DOPE) membranes in the order [MnW_6_O_24_]^8–^ > [Ni(OH)_6_W_6_O_18_]^4–^ > [TeW_6_O_24_]^6–^. The addition of negatively charged cardiolipin (CL) to DOPC reduced the interaction of POTs with the membrane. An opposite effect was observed for POMos, which changed the ζ-potential of neutral and slightly negatively charged membranes in the order [Al(OH)_6_Mo_6_O_18_]^3–^ > [Cr(OH)_6_Mo_6_O_18_]^3–^ >> [Ni(OH)_6_Mo_6_O_18_]^4–^. The addition of POMos increased the size of the liposomes in reverse order. The binding of [Al(OH)_6_Mo_6_O_18_]^3–^ to the PE-containing phospholipid membranes and the effect of ionic strength on the interaction of [Cr(OH)_6_Mo_6_O_18_]^3–^ with DOPC:CL liposomes could be inhibited by potassium fluoride (KF). Interestingly, KF did not inhibit the interaction of other POMos with membranes as indicated by ζ-potential measurements. These results suggest that the interaction of Anderson-Evans type POMs with phospholipid membranes is influenced more by their addenda and central ions than by their total charge. By unravelling the structure-activity relationships for the different POMs, we contribute to the design of biologically active POMs for therapeutic use.

## 1 Introduction

The family of inorganic anionic metal oxide clusters, polyoxometalates (POMs), includes numerous members with very distinct but highly adjustable structures. Due to their tuneable composition, variable structure and chemical properties, POMs have been successfully applied in various scientific fields (Guo et al., 2023). It is possible to incorporate different chemical ions into the metal-oxide framework of heteropolyoxoanions and even replace POM segments with organic ligands to form hybrid organic-inorganic POMs (Blazevic et al., 2015).

POMs have attracted considerable medical attention due to their antitumor, antiviral, and antibacterial activities, making them potential candidates for drug research and diagnostic applications (Bijelic et al., 2018; Bijelic et al., 2019; Aureliano et al., 2021; Lentink et al., 2023). POMs are active both at the cell surface (Inoue et al., 2005; Inoue et al., 2006) and in the cytoplasm (Inoue et al., 2005; Inoue et al., 2006). The degree of cellular penetration and localization of a drug directly affects its viral inhibition mechanism and other biological properties. An important advantage of POMs for medical applications is that many of their molecular properties, such as size and shape, surface charge distribution and acidity, can be modified to optimize the POMs for the recognition of biological target macromolecules and increase their reactivity (Rhule et al., 1998). The limitation associated with many POMs, such as those of the Keggin-, Wells-Dawson, or trivacant Keggin-derived sandwich archetype, when considered for medical applications, stems from their inherent inorganic nature, substantial molecular weight, and potential toxicity (Rhule et al., 1998).

**Figure 1:**
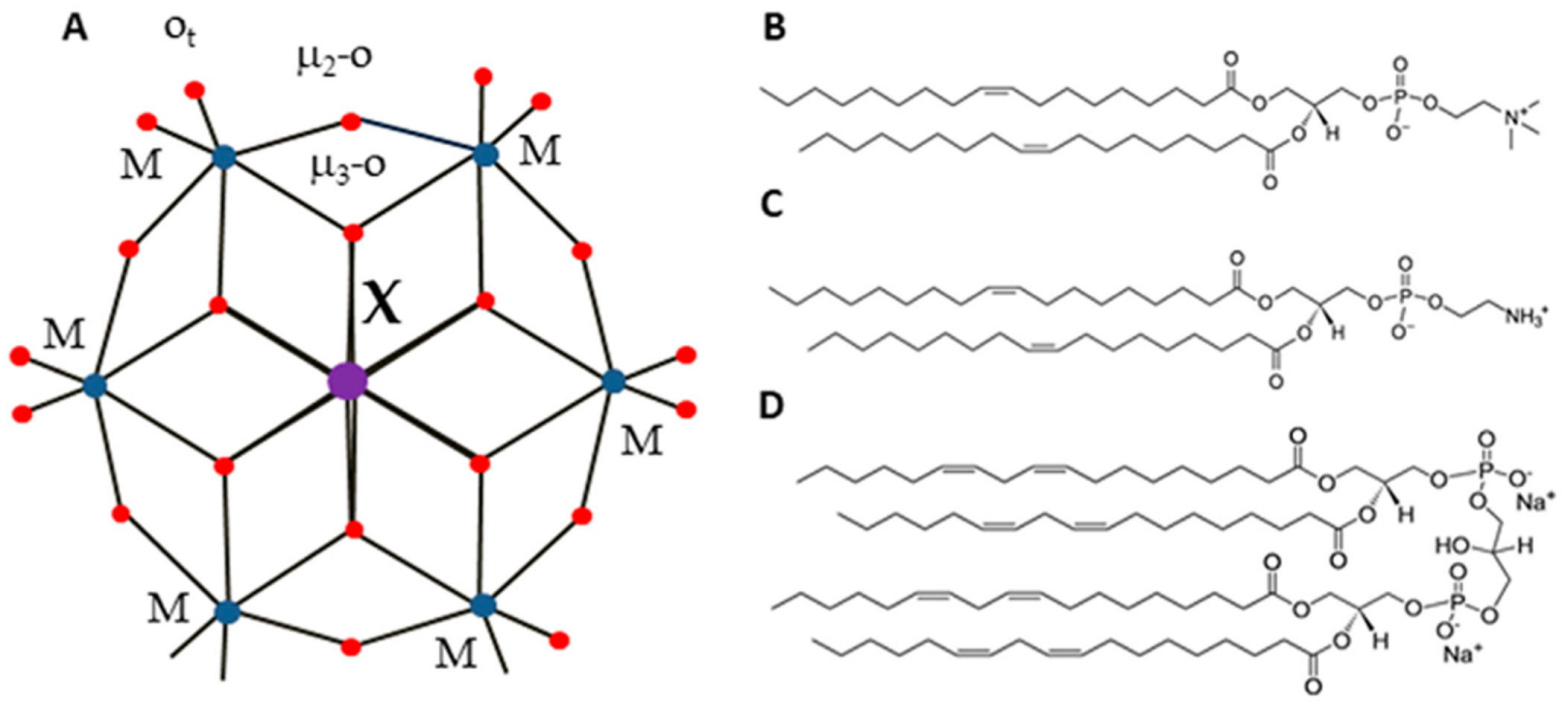
Structure of Anderson-Evans type POMs (A) and lipids (B–D). Color code: central ion, X (Al Ni, Mn, Sb, Te, or Cr) is in purple, addenda ions, M (W or Mo) are in blue. Oxygen, O, is in red. Lipids used in the study were: DOPC (B), DOPE (C) and cardiolipin CL, (D).

Recent studies have highlighted the profound impact that membrane interactions have on the biological activities of POMs. For instance, Kostenkova et al. (2023) found that specific polyoxidovanadates could interact with plasma membrane lipids, triggering aggregation and activation of G protein-coupled receptors (GPCRs) in CHO cells. This interaction not only affects receptor signaling but also modulates cellular responses such as cAMP levels, a key second messenger in cellular signaling pathways. Similarly, Samart et al. (2020) demonstrated that POMs’ ability to alter membrane lipid order correlates with changes in cellular processes and can lead to differences in cell viability and receptor activity. These findings suggest a direct link between the physicochemical properties of POMs at the membrane interface and their broader biological effects, underlining the relevance of our study which investigates how different POMs interact with lipid bilayers of varying compositions. By exploring these interactions, our research aims to shed light on the mechanisms through which POMs influence cellular behavior, potentially leading to novel therapeutic strategies that harness these interactions. Matsumoto et al. (2006) proposed that bacterial membranes contain a mosaic of microdomains of cardiolipin (CL) and phosphatidylethanolamine (DOPE) (Figures 1C, D), which are to a significant extent self-assembled according to their respective intrinsic chemical properties. The polar head group of the PE molecule contains both a cationic amine residue and an anionic phosphate residue. Each amine and unesterified phosphate oxygen can participate in two short-range intermolecular hydrogen bonds. Another membrane lipid, cardiolipin, has a double glycerophospholipid structure consisting of a glycerol residue and negatively charged phosphates in the head group.

In this study, we focus on the smallest POM heteropolyanion of the Anderson-Evans structure (Blazevic and Rompel, 2016), which can be readily tailored by varying the central heteroion, overcoming the limitations associated with larger POMs. The Anderson-Evans polyoxoanion has the general formula [H_y_(XO_6_)M_6_O_18_]^n-^, where y = 0–6, n = 2–8, M = addenda ion (Mo^VI^ or W^VI^), which are the main metal ions that complete the structure, and X = central hetero-ion in oxidation states from + 2 to + 7 (Anderson, 1937). The Anderson-Evans POM [XMo_6_O_24_]^n−^ contains an octahedral central ion surrounded by six {MoO_6_} or {WO_6_} octahedra via edge sharing (Blazevic and Rompel, 2016) (Figure 1A). The structure includes three types of oxygen ions: six triple-bridged oxygen ions (*μ*_3_-O), which link a heteroion to two addenda ions; six double-bridged oxygen ions (*μ*_2_-O), each connecting two addenda ions; and two terminal oxygen ions (O_t_) for each addenda ion, as shown in Figure 1. The oxidation state of a hetero-ion plays a significant role in the protonation mode of the triple-bridged oxygen ions (*μ*_3_-O) in the Anderson-Evans type POM (Sifaki et al., 2021). Furthermore, Anderson-Evans POMs exhibit exceptional stability under physiological pH conditions (Gumerova and Rompel, 2020; Gumerova and Rompel, 2023), a crucial feature for their potential applications in the biological field. The assessment of the biological activity of POMs requires an in-depth inorganic, biochemical, and biological approach (Gumerova and Rompel, 2021). Like other inorganic coordination compounds, POMs are flexible reactive molecules whose identity and integrity depend on the reaction conditions (pH, osmolarity, *etc*.) (Gumerova and Rompel, 2023).

We hypothesized that POMs of the Anderson-Evans archetype (i) can interact with a head group of PE and CL and (ii) that this interaction depends on the localization of POMs in the lipid membrane with respect to the lipid head group. The aim of this study was to characterize the interaction of different POMs of Anderson-Evans type with bilayer membranes of different lipid compositions relevant to the lipid composition of bacterial membranes.

## 2 Materials and methods

### 2.1 Chemicals

Na_2_SO_4_ (#8560), 2-(N-morpholino)ethanesulfonic acid (MES, #4256), tris(hydroxymethyl)-aminomethane (Tris, #AE15), chloroform (#AE54) were purchased from Carl Roth GmbH and Co. KG (Karlsruhe, Germany). NaF (#201154), 1,2-dioleoyl-sn-glycero-3-phosphocholine (DOPC, #P6354), 1,2-dioleoyl-sn-glycero-3-phosphoethanolamine (DOPE, #P1223) and cardiolipin (CL, #C0563), Na_2_WO_4_·2H_2_O, K_2_WO_4_, Na_2_MoO_4_·2H_2_O, AlCl_3_, Cr(NO_3_)_3_·9H_2_O, Ni(NO_3_)_2_·6H_2_O, KSb(OH)_6_ H_6_TeO_6_, MnSO_4_·H_2_O, Na_2_S_2_O_8_, HNO_3_, HCl were obtained from Sigma-Aldrich (Vienna, Austria) and used as received.

### 2.2 POMs synthesis

The POMs investigated in this work are presented in Table 1. Na_3_[Al(OH)_6_Mo_6_O_18_]·8H_2_O (Manikumari et al., 2002), Na_3_ [Cr(OH)_6_Mo_6_O_18_]·8H_2_O (Perloff, 1970), Na_4_ [Ni(OH)_6_Mo_6_O_18_]·16H_2_O (Gumerova et al., 2015), Na_4_ [Ni(OH)_6_W_6_O_18_]·16H_2_O (Rozantsev et al., 2009), K_5_ [H_2_SbMo_6_O_24_]·7H_2_O (Ogawa et al., 1988), K_5_[H_2_SbW_6_O_24_]·6H_2_O (Naruke and Yamase, 1992), Na_6_[TeMo_6_O_24_]·22H_2_O (Robl and Frost, 1993), Na_6_[TeW_6_O_24_]·22H_2_O (Schmidt et al., 1986), and Na_2_K_6_[MnW_6_O_24_]·12H_2_O (Nolan et al., 2000) were synthesized according to reported procedures (Table 1). and characterized with IR spectroscopy and proven latice constant (Supplementary Figure S4; Supplementary Table S1). POMs in powder form were dissolved in double-distilled H_2_O to obtain a stock solution of 5 or 10 mM. For the addition to the membrane POMs were dissolved in double-distilled H_2_O to achieve the required concentrations. All POMs are stable in the pH range of 6–7.5 which was proved by ESI-MS (Supplementary Figures S5, S6) and ^183^W (tungsten-183 isotope) NMR spectroscopy (Supplementary Figure S7).

### 2.3 FTIR and NMR spectroscopy

All FTIR spectra were recorded on a Bruker Vertex 70 IR spectrometer equipped with a single reflection diamond ATR unit. Frequencies are given in cm^−1^. Mass spectra were obtained with a timsTOF flex LC-MS system supplied by Bruker Daltonics Ltd. Bruker Daltonics Data Analysis 4.0 software was used to analyze the results. ^183^W NMR spectra were recorded on an Avance Neo 500 MHz FT-NMR spectrometer (Bruker, Rheinstetten, Germany) at 25°C. Chemical shifts were measured relative to 1 M Na_2_WO_4_. ^183^W NMR samples were prepared in 2.7 mL buffer with a POM concentration of 20 mM and measured in 10 mm tubes. The experimental time was approximately 60 h with a standard pulse program at 20.836 MHz and a flip angle of 63° with a relaxation delay of 1 s.

### 2.4 Preparation of unilamellar liposomes

Lipids were dissolved in chloroform, mixed in the required ratios (100 mol% DOPC, DOPC:CL 90:10, DOPC:DOPE 50:50) and evaporated under gaseous N_2_. 1 mL of buffer (20 mM Na_2_SO_4_, 10 mM Tris-HCl, 10 mM MES, pH 7.34) was added for a final lipid concentration of 0.4 mg/mL. Unilamellar liposomes were formed using a small volume extruder (#610023, Avanti Polar lipids, Alabaster, Alabama, United States) using 100 nm pore filters (#800309, Whatman, South Miami Ave, United States) or (AVESTIN, Europe, Mannheim, Germany) applied subsequently (Jovanovic et al., 2015). Liposomes were further diluted in buffer to a final concentration of 0.2 mg/mL.

### 2.5 ζ-potential measurements

The ζ-potential (Φ_ζ_) and size of liposomes were measured using a Zetasizer Nano (ZS ZEN3600, Malvern Panalytical Ltd., United Kingdom) as previously described (Pashkovskaya et al., 2018). The velocity of liposome movement in an electric field was derived from the Doppler shift of a scattered laser beam. From these data the electrophoretic mobility of the liposomes was determined and the Smoluchowski model (Hunter, 1981) was used to calculate Φ_ζ_. The Zetasizer derives a particle size from the Brownian motion of the particles measured by dynamic light scattering (DLS). All measurements were performed at room temperature T = 25°C and pH = 7.34.

### 2.6 Statistics

Data analysis and fitting of size and ζ-potential measurements were performed using Sigma Plot 12.0 (Systat Software GmbH, Erkrath, Germany) and are presented as the mean ± SD of at least three independent measurements.

## 3 Results

To evaluate the effect of POMs on the membrane, we measured the ζ-potential of the liposomes both in the presence and in the absence (control) of the investigated POMs (see Methods). Taken into account the lipid composition of bacterial membranes (Matsumoto et al., 2006), we produced liposomes with three different types of lipid configurations: (i) neutral DOPC, (ii) a mixture of DOPC and DOPE in a 50:50 mol% ratio, and (iii) negatively charged DOPC combined with CL in a 90:10 mol% ratio (Figures 1B–D). We also investigated the sizes of the liposomes in the presence of Anderson-Evans type POMs (Figure 1; Table 1).

### 3.1 Effect of POMs on neutral membrane made of DOPC

**Figure 2:**
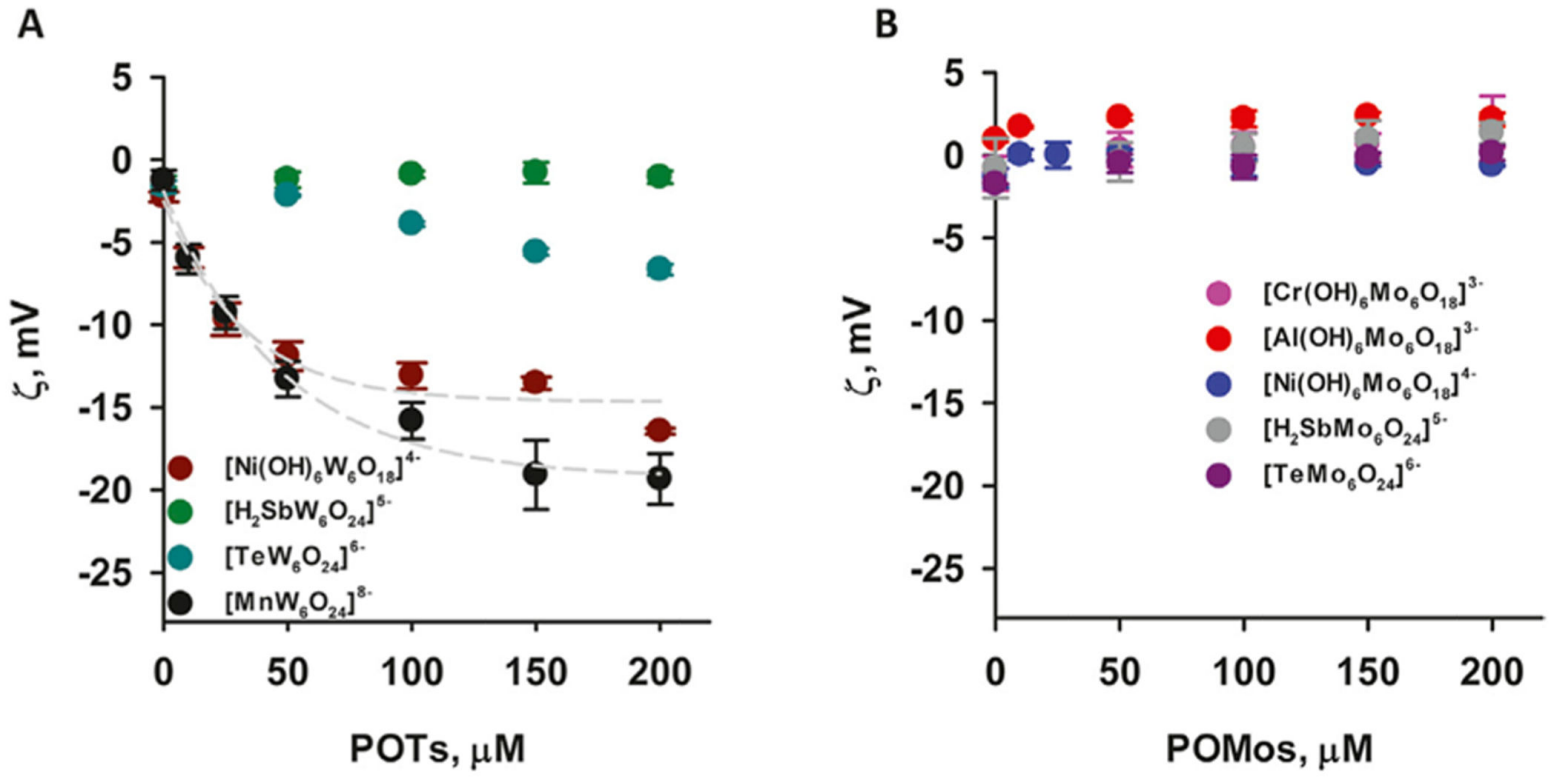
ζ-Potential of neutral liposomes in the presence of POTs (A) and POMos (B). The liposomes were prepared from DOPC. The lipid concentration was 0.2 mg/mL. The buffer solution consisted of 20 mM Na_2_SO_4_, 10 mM MES, 10 mM Tris-HCl at pH = 7.34 and T = 25°C.

To understand the role of charge and hydrophobicity of POMs on their adsorption to the membrane, we first studied their interaction with the neutral (DOPC) membrane. While most bacterial membranes are predominantly composed of negatively charged or zwitterionic lipids, some bacteria contain neutral lipids. The use of DOPC allows us to study how neutral lipids in bacterial membranes might interact with POMs without the complicating factor of charge. We measured the ζ-potential of liposomes and changes in liposome size throughout the adsorption process of the four polyoxomolybdates (POMos) and five polyoxotungstates (POTs), respectively (Figure 2). Within each group, we compared the POMs containing different central ions with varying charges. The three polyoxotungstates (POTs) ([MnW_6_O_24_]^8–^ [TeW_6_O_24_]^6–^ and [Ni(OH)_6_W_6_O_18_]^4–^) changed the ζ-potential of liposomes exponentially depending on the concentration of the respective POT (Figure 2A). While [MnW_6_O_24_]^8–^ showed a slightly higher affinity for liposome adsorption compared to [Ni(OH)_6_W_6_O_18_]^4−^, the degree of adsorption does not correlate well with the substantial difference in charge between these two POTs (Figure 2A). To investigate whether the ζ-potential depends on addenda ion type, we compared POMs with similar central ion (Te), but different addenda ion. The addition of [TeW_6_O_24_]^6–^ resulted in a slight decrease of the ζ-potential (Figure 2A), whereas [TeMo_6_O_24_]^6–^ did not affect it (Figure 2B). In addition, the size of the liposomes increased by 10% upon addition of [TeW_6_O_24_]^6–^ (Table 2A). No change in ζ-potential was observed in DOPC liposomes after addition of [Al(OH)_6_Mo_6_O_18_]^3–^, [Cr(OH)_6_Mo_6_O_18_]^3–^, [Ni(OH)_6_Mo_6_O_18_]^4–^, [H_2_SbMo_6_O_24_]^5–^ (Figure 2B), and [H_2_SbW_6_O_24_]^5–^ (Figure 2A), although their charges were similar to or less than those of [Ni(OH)_6_W_6_O_18_]^4–^ (Figure 2A) and [MnW_6_O_24_]^8–^ (Figure 2A), which affected the ζ-potential. No significant change (less than 10%) in the size of DOPC liposomes was observed after the addition of Anderson-type POMs, except for [Al(OH)_6_Mo_6_O_18_]^3–^ and [Cr(OH)_6_Mo_6_O_18_]^3–^ (Table 2A). After the addition of 200 µM [Al(OH)_6_Mo_6_O_18_]^3–^, two distinct size peaks were observed at 194.78 ± 17.77 and 2093.43 ± 105.04 nm, compared to the size of liposomes without [Al(OH)_6_Mo_6_O_18_]^3–^, which was 113.83 ± 7.72 nm. Similarly, the addition of 200 µM [Cr(OH)_6_Mo_6_O_18_]^3–^ to DOPC liposomes resulted in a doubling of their size. These changes may be due to aggregation, fusion, or both, of the POM-containing liposomes. Notably, [Al(OH)_6_Mo_6_O_18_]^3–^ and [Cr(OH)_6_Mo_6_O_18_]^3–^ did not alter the ζ-potential of the neutral liposomes, but significantly increased their size.

### 3.2 Effect of POMs on slightly negative membranes containing DOPE and more negatively charged membranes containing cardiolipin

**Figure 3:**
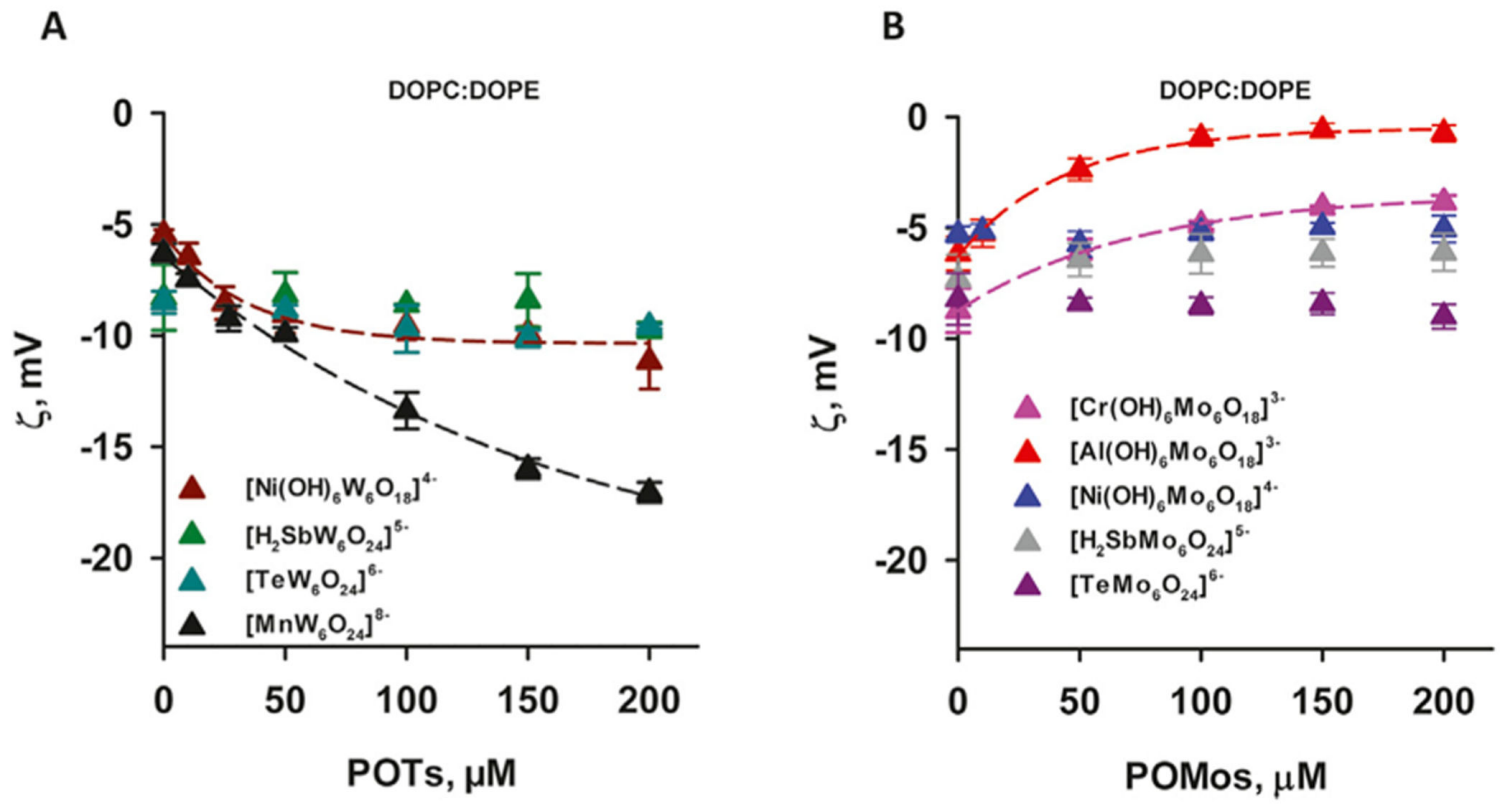
ζ-Potential of slightly negatively charged liposomes containing POTs (A) and POMos (B). The liposomes were prepared from DOPC:DOPE (50:50%). The lipid concentration was 0.2 mg/mL. The buffer solution consisted of 20 mM Na_2_SO_4_, 10 mM MES, 10 mM Tris-HCl at pH = 7.34 and T = 25°C.

Most of *alpha, beta, gamma*, and *delta* proteobacteria accumulate two major membrane phospholipids: the predominant zwitterionic phospholipid is PE, whereas the cardiolipin is anionic in nature (Sohlenkamp and Geiger, 2016). To evaluate the influence of the negative membrane charge on the absorption of POMs we formed liposomes with the lipid composition DOPC:DOPE and DOPC:CL. Under the experimental conditions used (20 mM Na_2_SO_4_, 10 mM MES, 10 mM Tris-HCl and pH 7.34), the DOPC:DOPE liposomes were negatively charged (-(5-7) mV, Figure 3). The adsorption of two polyoxotungstates [MnW_6_O_24_]^8–^ and [Ni(OH)_6_W_6_O_18_]^4–^ onto DOPC:DOPE liposomes shifted the ζ-potential towards more negative values (Figure 3A), similar to those observed for DOPC liposomes. In contrast, with two POMos carrying a charge of −3 ([Al(OH)_6_Mo_6_O_18_]^3–^ and [Cr(OH)_6_Mo_6_O_18_]^3–^), we observed a different effect: the ζ-potential of the liposomes approached zero as the concentration of POMos increased (Figure 3B). Notably, we also observed the increase in liposome size in the presence of [MnW_6_O_24_]^8–^ [Cr(OH)_6_Mo_6_O_18_]^3–^, and especially [Al(OH)_6_Mo_6_O_18_]^3–^ (Supplementary Figures S1A, C, D; Table 2B) [H_2_SbMo_6_O_24_]^5–^ did not significantly alter the ζ -potential of DOPC: DOPE liposomes (Figure 3A). [H_2_SbW_6_O_24_]^5-^, [TeMo_6_O_24_]^6–^, and [Ni(OH)_6_Mo_6_O_18_]^4–^ did not interact with DOPC:DOPE liposomes (Figure 3; Table 2B).

**Figure 4:**
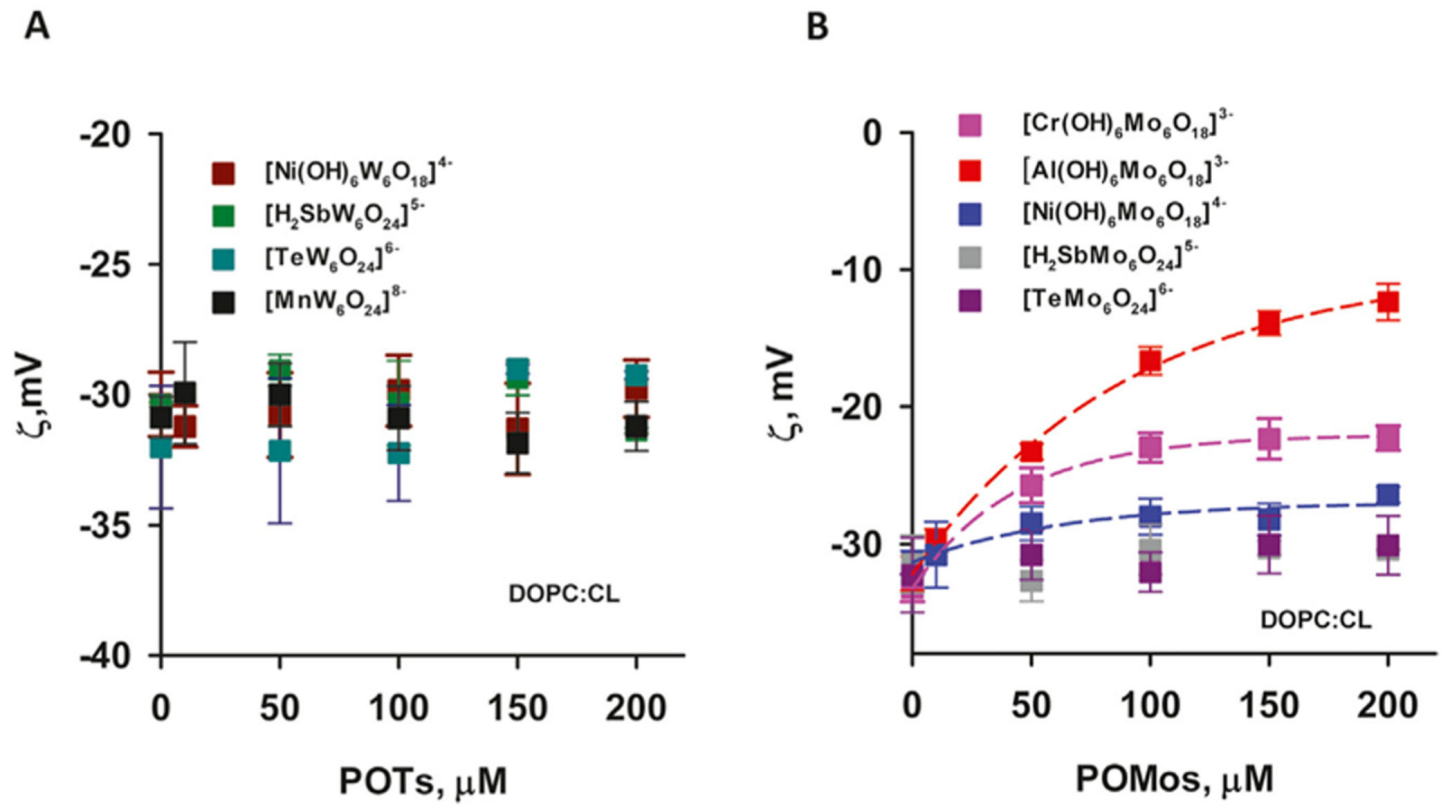
ζ-Potential negatively charged liposomes containing POTs (A) and POMos (B). The liposomes were prepared from DOPC:CL (90:10%). The lipid concentration was 0.2 mg/mL. The buffer solution consisted of 20 mM Na_2_SO_4_, 10 mM MES, 10 mM Tris-HCl at pH = 7.34 and T = 25°C.

**Figure 5:**
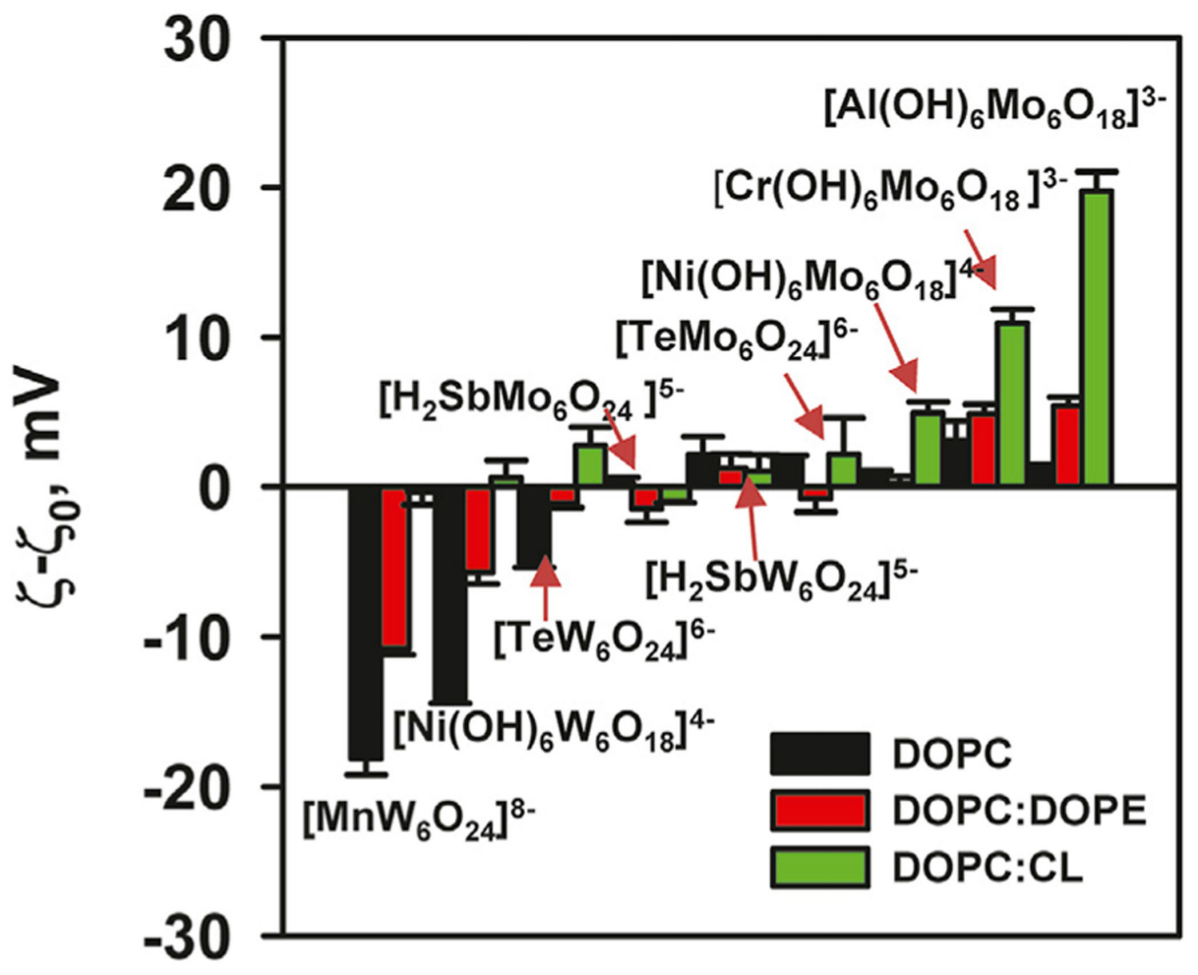
ζ-Potential of liposomes in the presence of POMs. Liposomes were prepared from DOPC, DOPC:DOPE (50:50%) and DOPC:CL (90: 10%). The lipid concentration was 0.2 mg/mL and the POMs concentration was 200 mM. The buffer solution consisted of 20 mM Na_2_SO_4_, 10 mM MES, 10 mM Tris-HCl at pH = 7.34 and T = 25°C.

The effect of [Al(OH)_6_Mo_6_O_18_]^3–^ on liposomes prepared from DOPC:CL was particularly pronounced. Its adsorption increased the potential from (−32.14 ± 1.26) mV to (−12.37 ± 1.32) mV (Figure 4B) and increased the size of the liposomes (Table 2C). Conversely, for [Cr(OH)_6_Mo_6_O_18_]^3–^, the ζ-potential of the liposomes increased from (−33.2 ± 1.02) mV to (−22.28 ± 0.89) mV, while for [Ni(OH)_6_Mo_6_O_18_]^4–^ we measured an increase from (−31.36 ± 0.88) mV to (−26.40 ± 0.58) mV. The ζ–potential change was exponential for all three POMos. However, the size of the liposomes remained unchanged for [Cr(OH)_6_Mo_6_O_18_]^3–^ and [Ni(OH)_6_Mo_6_O_18_]^4–^ (Supplementary Figure S3; Table 2C). Other POMs did not interact with the negatively charged liposomes of DOPC:CL (Figure 4; Table 2C). Figure 5 shows that there is no charge dependence among all nine POMs studied.

The presence of W ions in the POTs facilitated their interaction with DOPC liposomes, leading to an increase in the negative potential of the liposomes. Conversely, this effect was not observed for POMos. On the other hand, POMos containing Mo neutralized the charge of the negative liposomes of DOPC:CL and DOPC:PE. This suggests that other types of interactions may be involved.

### 3.3 Influence of ionic strength on the interaction of POMos with negatively charged liposomes

Figure 5 shows that only POMos induce a shift in the ζ-potential of DOPC:CL liposomes. Interestingly, this effect does not seem to depend on the charge of the liposomes. To understand the nature of this interaction, we measured the dependence of the ζ-potential of DOPC:CL liposomes on the ionic strength of the buffer solution in the presence of POMos.

**Figure 6:**
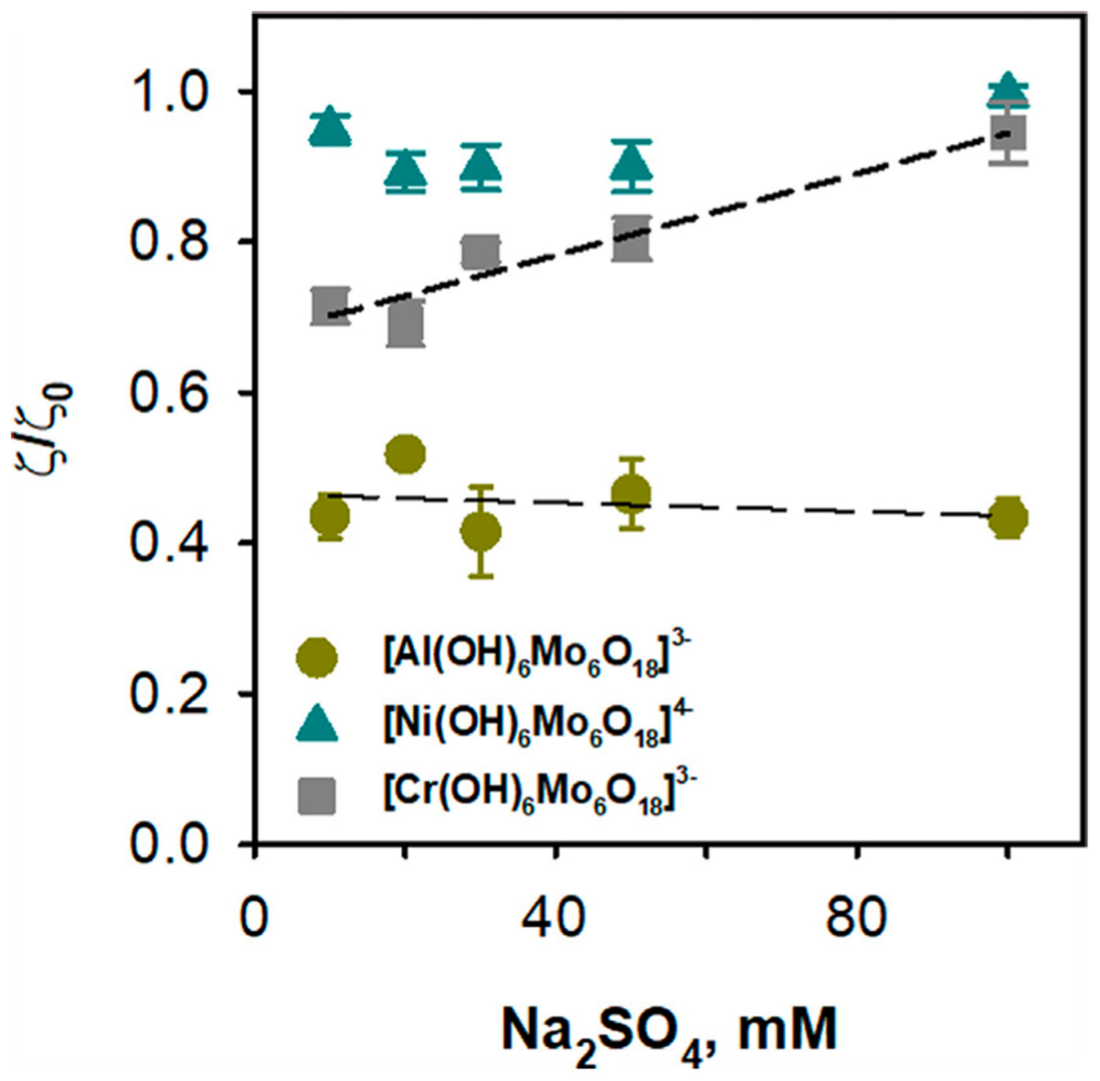
Effect of ionic strength on the binding of POMos to negatively charged liposomes. The liposomes were prepared from DOPC:CL (90: 10%) and the lipid concentration was 0.2 mg/mL. The buffer solution consisted of 10, 20, 30, 50 and 100 mM Na_2_SO_4_, 10 mM MES, 10 mM Tris-HCl at pH = 7.34 and T = 25°C. The concentration of POMos was 100 µM.

The change in ionic strength itself affected the charge of DOPC:CL liposomes due to the negatively charged head group of cardiolipin. Increasing the ionic strength resulted in a loss of the negative charge of the liposomes in the control (Supplementary Figure S3). Figure 6 and Supplementary Figure S3 show that [Al(OH)_6_Mo_6_O_18_]^3–^, and [Cr(OH)_6_Mo_6_O_18_]^3–^, in contrast to [Ni(OH)_6_Mo_6_O_18_]^4-^, mitigate the effect of ionic strength in DOPC:CL liposomes without POMos. The comparison of the relative effect of the interaction of [Al(OH)_6_Mo_6_O_18_]^3–^ with DOPC:CL liposomes in different media suggest that the ionic strength had no effect on this interaction (Figure 6). Since the difference between [Al(OH)_6_Mo_6_O_18_]^3–^ and [Cr(OH)_6_Mo_6_O_18_]^3–^ is only in the central heteroion, a new question arises as to the reason for such a strong interaction between [Al(OH)_6_Mo_6_O_18_]^3–^ and negatively charged liposomes.

### 3.4 Influence of ionic strength on the interaction of Anderson-Evans POTs with DOPE-containing liposomes

**Figure 7:**
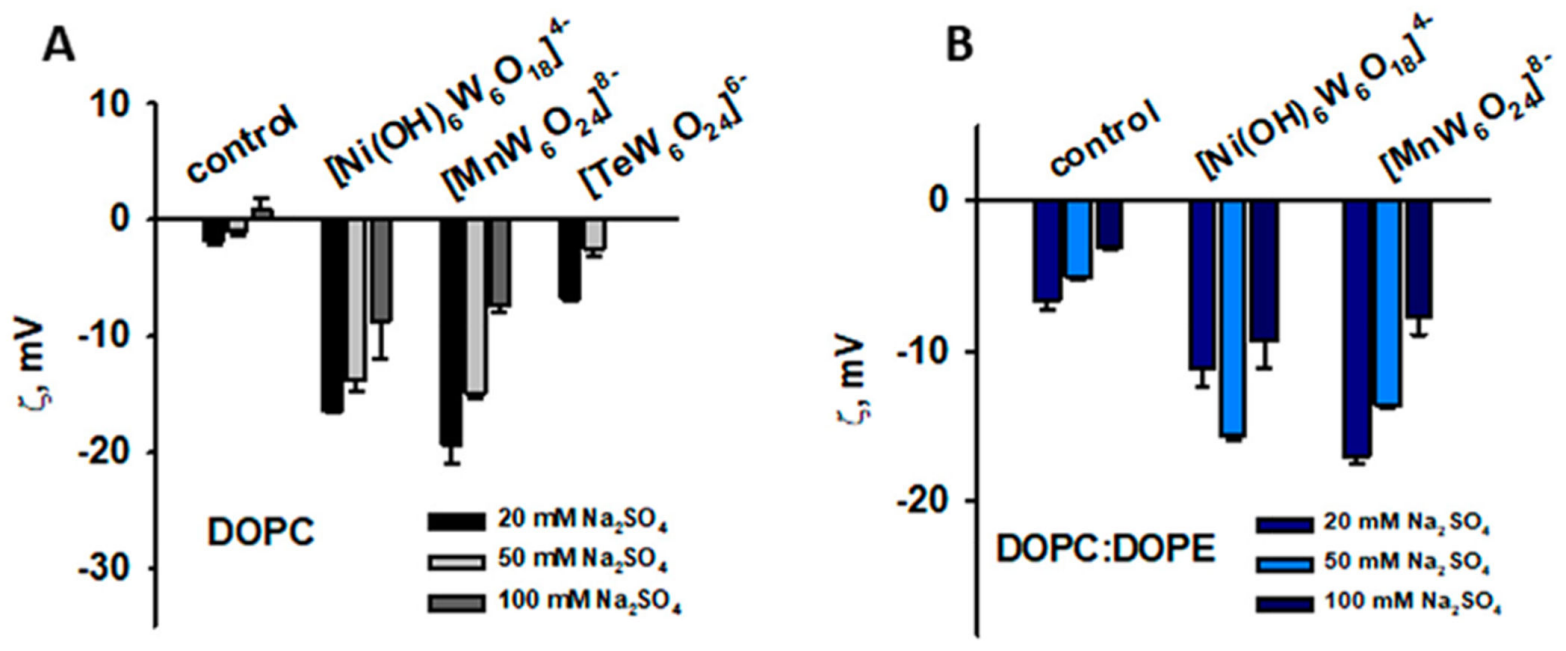
Influence of ionic strength on the binding of POTs to neutral liposomes prepared from DOPC (A) and negatively charged liposomes prepared from DOPC:DOPE (50:50%) (B). The lipid concentration was 0.2 mg/mL and the POTs concentration was 200 mM. The buffer solution consisted of 20, 50 and 100 mM Na_2_SO_4_, 10 mM MES, 10 mM Tris-HCl at pH = 7.34 and T = 25°C.

To determine the role of electrostatic interactions in the binding of polyoxotungstates (POTs) to DOPE-containing liposomes, experiments were performed under different ionic strength conditions. The addition of [MnW_6_O_24_]^8-^ to DOPC and DOPC:DOPE liposomes and [Ni(OH)_6_W_6_O_18_]^4-^ or [TeW_6_O_24_]^6–^ to DOPC liposomes resulted in the same shielding effect observed in the control (Figures 7A,B). However, this effect was not observed in DOPC:DOPE liposomes treated with [Ni(OH)_6_W_6_O_18_]^4–^ (Figure 7B). A possible explanation is that the POTs either desorb from the liposomes or are located deeper within the lipid tails.

### 3.5 Effect of potassium fluoride on the interaction of POMos with DOPC:CL and DOPC:PE liposomes

**Figure 8:**
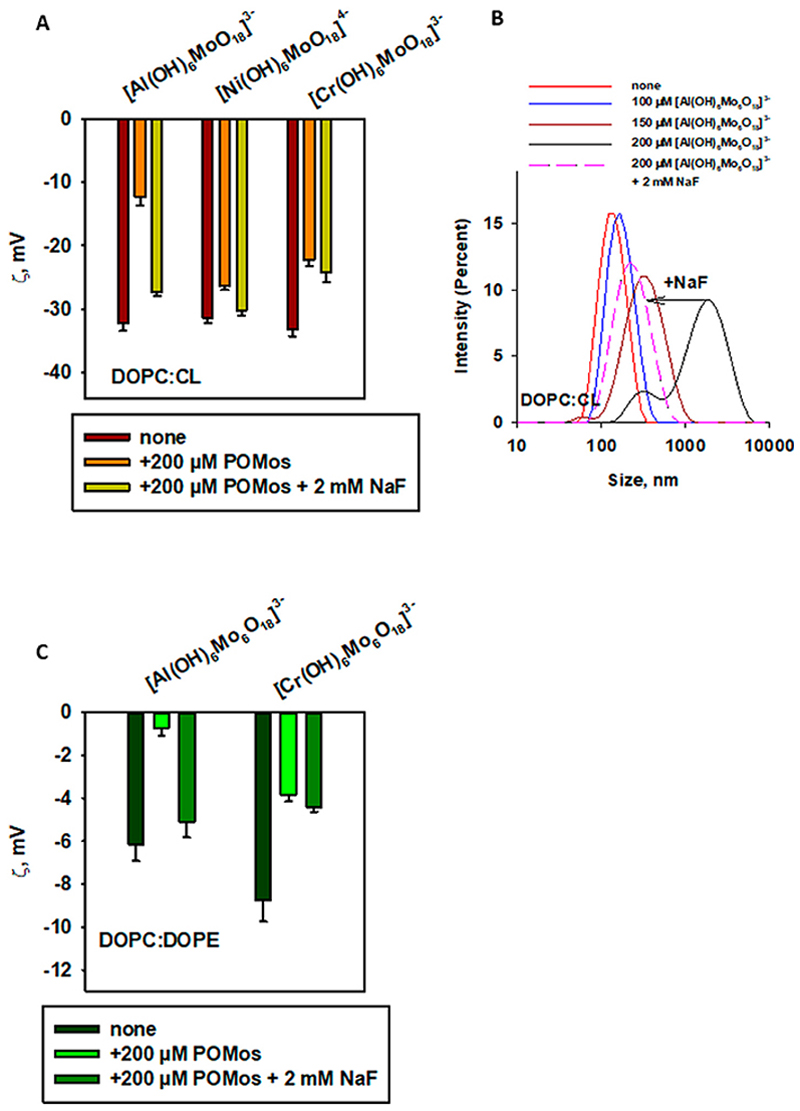
Effect of fluoride on the binding of POMos to negatively charged liposomes, measured by ζ-potential (A, C) or liposome size distribution by intensity (B). Liposomes were prepared from DOPC:CL (90:10%, (A, B) or DOPC:DOPE (50:50% in (C). The lipid concentration was 0.2 mg/mL. The buffer solution consisted of 20 mM Na_2_SO_4_, 10 mM MES, 10 mM Tris-HCl at pH = 7.34 and T = 25°C.

To understand what kind of interactions other than electrostatic might be involved in the interaction between [Al(OH)_6_Mo_6_O_18_]^3–^ and DOPC:CL, we referred to our data showing fluoride-induced inhibition of the binding of aluminium phthalocyanines to artificial (Rokitskaya et al., 2000; Pashkovskaya et al., 2007) and natural (Ben-Hur et al., 1993) membranes, as well as to proteins (Ben-Hur et al., 1991). We hypothesized that POMos containing a central Al(III) ion might also be sensitive to fluoride. Therefore, we tested three POMos ([Al(OH)_6_Mo_6_O_18_]^3–^, [Cr(OH)_6_Mo_6_O_18_]^3–^, and [Ni(OH)_6_Mo_6_O_18_]^4–^) that altered the ζ-potential of negatively charged liposomes (prepared from DOPC:CL and DOPC:DOPE). We observed an inhibitory effect of fluoride on the [Al(OH)_6_Mo_6_O_18_]^3–^ binding to the membrane, a small to no inhibitory effect on binding of [Ni(OH)_6_Mo_6_O_18_]^4–^, and no effect on binding of [Cr(OH)_6_Mo_6_O_18_]^3-^ (Figure 8A). Interestingly, the size of the liposomes was also restored under the effect of fluoride in the case of [Al(OH)_6_Mo_6_O_18_]^3–^ (Figure 8B). The same effect of fluoride was observed on the liposomes prepared from DOPC: DOPE in the presence of [Al(OH)_6_Mo_6_O_18_]^3–^: the ζ-potential was restored (Figure 8C), the liposome size was also restored, but not to the original value (Supplementary Figure S1C). In the case of DOPC:DOPE liposomes supplemented with 200 µM [Cr(OH)_6_Mo_6_O_18_]^3–^, no effect of fluoride was observed (Figure 8C; Supplementary Figure S1D).

## 4 Discussion

All POMs interact uniquely with membranes depending on their lipid composition. There is no charge dependence in the interaction of all investigated POMs with different types of lipid membranes.

However, a difference is observed between POMs containing Mo and W addenda (Figure 5). The interaction of POTs with the neutral membrane (DOPC) resulted in a negative membrane charge, whereas their interaction with DOPC:DOPE membranes, which have a small negative membrane charge resulted in a large negative surface potential. In contrast, POTs did not interact with a negatively charged (DOPC:CL) membrane, nor did we observe any change in liposome size or ζ-potential.

Anderson-Evans type POMs may be more deeply embedded in the lipid tails, thus not affecting the ζ-potential of liposomes. POMos are more hydrophilic and may localize near the lipid heads, influencing the negative ζ-potential of liposomes. This interaction appears to be non-specific for [Cr(OH)_6_Mo_6_O_18_]^3–^ and [Ni(OH)_6_Mo_6_O_18_]^4–^ (Figures 6, 8A; Table 2). However [Al(OH)_6_Mo_6_O_18_]^3-^ interacts specifically with charged lipid membranes or the head of phospholipids, and this interaction is not disrupted by ionic force (Figure 6) but is inhibited by fluoride (Figure 8).

Comparing the effect of three POTs ([MnW_6_O_24_]^8–^, [TeW_6_O_24_]^6–^, and [Ni(OH)_6_W_6_O_18_]^4–^) on the ζ-potential of neutral liposomes, we see no correlation between the charge of the POTs and the ζ-potential of the membrane (Figure 5). There is also no correlation in the interaction of these POTs with the slightly negative membrane DOPC:DOPE (Figure 5). Similarly, the interactions of POMos ([Al(OH)_6_Mo_6_O_18_]^3–^, [Cr(OH)_6_Mo_6_O_18_]^3–^, and [Ni(OH)_6_Mo_6_O_18_]^4–^) with negative and slightly negative membranes do not correlate with the POMos charge (Figure 5).

The addenda ions in Anderson-Evans POMs significantly influence the hydrophobicity. POTs are more hydrophobic than POMos, which determines the interaction and localization of POMs in different types of lipid membranes. In POMos, a central heteroion also plays a major role in the interaction of POMos with negatively charged membranes, leading to a change in their ζ-potential. If we separate and compare our POM series with W and Mo additions, a clear pattern emerges (Figure 5). Anderson-Evans polyoxotungstates alter the ζ-potential when interacting with liposomes containing neutral phospholipids but have no effect on negatively charged phospholipids (Figures 2A, 4A). Conversely, Anderson-Evans polyoxomolybdates alter the ζ-potential when interacting with liposomes containing negative phospholipids, but not with those containing neutral phospholipids (Figures 2B, 4B).

The central ion in Mo-containing Anderson-Evans POMs contributes to the interaction with phospholipid membranes [Al(OH)_6_Mo_6_O_18_]^3–^, [Cr(OH)_6_Mo_6_O_18_]^3–^, and [Ni(OH)_6_Mo_6_O_18_]^4–^ are examples of POMos that altered the ζ-potential of negatively charged liposomes (Figures 3B, 4B). Notably, two of them share the same charge and addenda ions, but differ in their central ion, which affects the efficacy and nature of their interactions with phospholipid membranes. Specifically, fluoride affects the binding of [Al(OH)_6_Mo_6_O_18_]^3-^ to phospholipid liposomes, but does not impact the binding of [Cr(OH)_6_Mo_6_O_18_]^3-^ nor [Ni(OH)_6_Mo_6_O_18_]^3-^ (Figure 8).

We assumed that in the case of [Al(OH)_6_Mo_6_O_18_]^3–^ the formation of the coordination bond of the positively charged central Al^3+^ with fluoride could be facilitated by Coulomb attraction, as discussed in our previous publication (Pashkovskaya et al., 2007) for tetrasulfonated phthalocyanines of aluminum, due to the coordination ability of aluminum in complexes. Whether this ability of aluminum leads to a specific interaction of [Al(OH)_6_Mo_6_O_18_]^3–^ with membranes containing phospholipids is still an open question.

The interactions of POMs with lipid membranes, as evidenced by their distinct binding patterns to different lipid compositions, underscore their specific biological activities and potential therapeutic implications. These interactions are intricately tailored to the structural characteristics of the lipid bilayers and are not uniform across all types. For instance, certain POMs show a selective affinity for membranes with specific lipid compositions, leading to changes in membrane fluidity and integrity. This selectivity might influence cellular processes such as receptor signaling, ion transport, and enzyme activity, directly linking the physicochemical properties of POMs to critical biological outcomes (Althumairy et al., 2020; Samart et al., 2020; Kostenkova et al., 2021; Kostenkova et al., 2023). Moreover, the ability of some POMs to alter the ζ-potential of the lipid membrane suggests that they can act as indirect modifiers or regulators of the properties of membrane proteins, enzymes, transporters, and channels. This modulation can be reversible and is sometimes regulated by factors such as ionic strength or the presence of fluoride. Such findings suggest that the molecular architecture of POMs, combined with the lipid composition of target membranes, may dictate the biological pathways affected by these interactions, potentially guiding the development of POM-based therapies targeting specific cellular dysfunctions.

## Supplementary Material

Supplementary Material

## Figures and Tables

**Table 1:** Properties of the Anderson-type POMs.

Formula	M,g/mol	Charge	References
Na_3_[Al(OH)_6_Mo_6_O_18_]·8H_2_O	1,205.75	−3	Manikumari et al. (2002)
Na_3_[Cr(OH)_6_Mo_6_O_18_]·8H_2_O	1,230.76	−3	Perloff (1970)
Na_4_[Ni(OH)_6_Mo_6_O_18_]·16H_2_O	1,404.33	−4	Gumerova et al. (2015)
Na_4_[Ni(OH)_6_W_6_O_18_]·16H_2_O	1,931.73	−4	Rozantsev et al. (2009)
K_5_[H_2_SbMo_6_O_24_]·7H_2_O	1,405.00	−5	Ogawa et al. (1988)
K_5_[H_2_SbW_6_O_24_]·6H_2_O	1,914.38	−5	Naruke and Yamase (1992)
Na_6_[TeMo_6_O_24_]·22H_2_O	1,621.50	−6	Robl and Frost (1993)
Na_6_[TeW_6_O_24_]·22H_2_O	2,148.56	−6	Schmidt et al. (1986)
Na_2_K_6_[MnW_6_O_24_]·12H_2_O	2,038.53	−8	Nolan et al. (2000)

**Table 2:** Average size of liposomes composed of DOPC (A) or DOPC:DOPE (B) in the presence and absence of Anderson-type POMs.

Lipid	POMs structure	Charge	Size of the pure liposomes, nm	Liposome size in the presence of 200 μM POMs, nm	Size increasing, %
A					
DOPC	Na_3_[Al(OH)_6_Mo_6_O_18_]·8H_2_O	−3	113.83 ± 7.72	**194.78 ± 17.77 and 2,093.43 ± 105.04**	**71.1 and 1,738.7**
	Na_3_[Cr(OH)_6_Mo_6_O_18_]·8H_2_O	−3	129.45 ± 3.45	**235.28 ± 8.48**	**81.8**
	Na_4_[Ni(OH)_6_Mo_6_O_18_]·16H_2_O	−4	135.30 ± 1.94	133.03 ± 0.51	-
	Na_4_[Ni(OH)_6_W_6_O_18_]·16H_2_O	−4	125.87 ± 2.99	125.64 ± 1.55	-
	K_5_[H_2_SbMo_6_O_24_]·7H_2_O	−5	130.00 ± 2.10	127.94 ± 1.56	-
	K_5_[H_2_SbW_6_O_24_]·6H_2_O	−5	128.70 ± 0.30	133.63 ± 0.47	-
	Na_6_[TeMo_6_O_24_]·22H_2_O	−6	124.25+0.85	128.34 ± 0.92	-
	Na_6_[TeW_6_O_24_]·22H_2_O	−6	129.00 ± 2.79	142.07 ± 5.97	10.1
	Na_2_K_6_[MnW_6_O_24_]·12H_2_O	−8	125.50 ± 2.0	127.09 ± 0.99	-
B					
DOPC: DOPE	Na_3_[Al(OH)_6_Mo_6_O_18_]·8H_2_O	−3	129.67 ± 1.27	**3,127.06 ± 224.54**	**2,311.6**
	Na_3_[Cr(OH)_6_Mo_6_O_18_]·8H_2_O	−3	125.67 ± 0.57	**279.20±54.40 and 1,223.50 ± 195.50**	**122.2 and 873.6**
	Na_4_[Ni(OH)_6_Mo_6_O_18_]·16H_2_O	−4	133.65 ± 2.05	134.48 ± 1.43	-
	Na_4_[Ni(OH)_6_W_6_O_18_]·16H_2_O	−4	132.87 ± 4.35	142.97 ± 2.95	-
	K_5_[H_2_SbMo_6_O_24_]·7H_2_O	−5	126.90 ± 2.02	133.36 ± 2.72	-
	K_5_[H_2_SbW_6_O_24_]·6H_2_O	−5	122.90 ± 1.90	126.32 ± 1.26	-
	Na_6_[TeMo_6_O_24_]·22H_2_O	−6	125.80 ± 3.03	129.85 ± 1.73	-
	Na_6_[TeW_6_O_24_]·22H_2_O	−6	125.80 ± 0.40	128.55 ± 1.64	-
	Na_2_K_6_[MnW_6_O_24_]·12H_2_O	−8	127.60 ± 4.48	**186.79 ± 6.35**	**46.4**
C					
DOPC: CL	Na_3_[Al(OH)_6_Mo_6_O_18_]·8H_2_O	−3	110.86 ± 7.74	**265.20 ± 59.23 and 1928.33 ± 263.73**	**139.2 and 1,639.4**
	Na_3_[Cr(OH)_6_Mo_6_O_18_]·8H_2_O	−3	118.93 ± 0.64	132.08 ± 1.62	11.1
	Na_4_[Ni(OH)_6_Mo_6_O_18_]·16H_2_O	−4	121.15 ± 0.76	120.85 ± 0.87	-
	Na_4_[Ni(OH)_6_W_6_O_18_]·16H_2_O	−4	123.50 ± 1.01	124.23 ± 0.80	-
	K_5_[H_2_SbMo_6_O_24_]·7H_2_O	−5	120.00 ± 0.20	119.15 ± 0.24	-
	K_5_[H_2_SbW_6_O_24_]·6H_2_O	−5	120.55 ± 0.55	119.98 ± 0.42	-
	Na_6_[TeMo_6_O_24_]·22H_2_O	−6	121.90 ± 2.00	121.10 ± 0.72	-
	Na_6_[TeW_6_O_24_]·22H_2_O	−6	123.35 ± 0.65	122.66 ± 0.58	-
	Na_2_K_6_[MnW_6_O_24_]·12H_2_O	−8	101.22 ± 10.24	125.73 ± 1.92	24.2

## Data Availability

The raw data supporting the conclusions of this article will be made available by the authors, without undue reservation.
