# Supplementary material for: Molecular interactions at the interface: polyoxometalates of the Anderson-Evans type and lipid membranes
Source: Front Chem Biol. Author manuscript; Available in PMC 2025 May 10. (PMC7617656; doi:10.3389/fchbi.2024.1454558)
Supplement: Supplementary Material [file EMS204739-supplement-Supplementary_Material.PDF]

Molecular interactions at the interface: polyoxometalates of the Anderson-Evans type and lipid membranes

Alina A. Pashkovskaya[1], Nadiia I. Gumerova[2], Annette Rompel[2], Elena E. Pohl[1]

[1]Physiology and Biophysics, Department of Biological Sciences and Pathobiology, University of Veterinary Medicine, 1210 Vienna, Austria; elena.pohl@vetmeduni.ac.at; alina.pashkovskaya@vetmeduni.ac.at

[2]Universität Wien, Fakultät für Chemie, Institut für Biophysikalische Chemie, 1090 Wien, Austria; nadiia.gumerova@univie.ac.at; annette.rompel@univie.ac.at; http://www.bpc.univie.ac.at

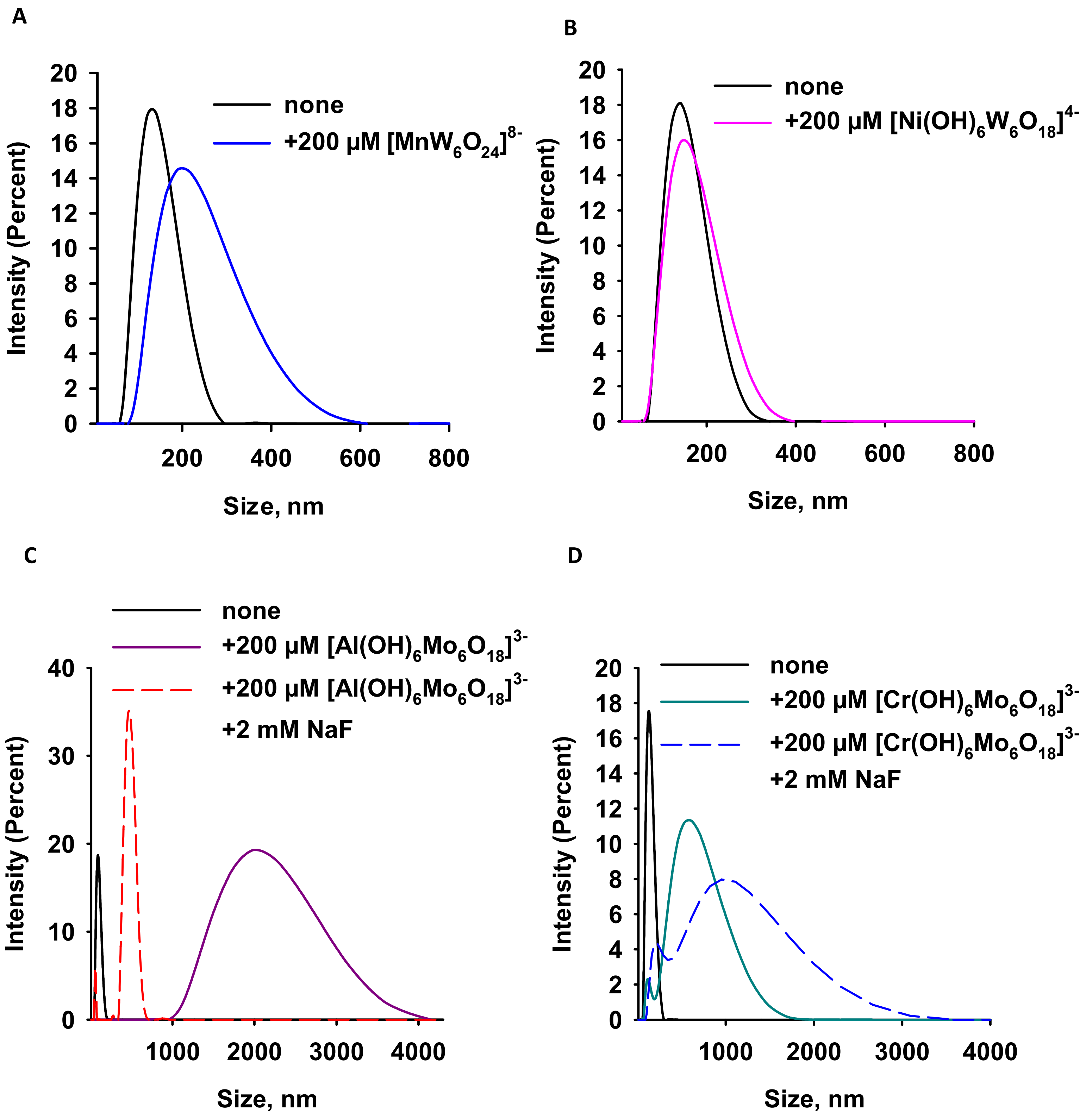

Supplementary Figure 1. Size distribution of liposomes with and without 200 μM POMs. The liposomes were prepared from DOPC:DOPE (50:50%). The lipid concentration was 0.2 mg/ml. The buffer solution consisted of 20 mM Na_2SO_4, 10 mM MES, 10 mM Tris at pH=7.34 and T=25°C.

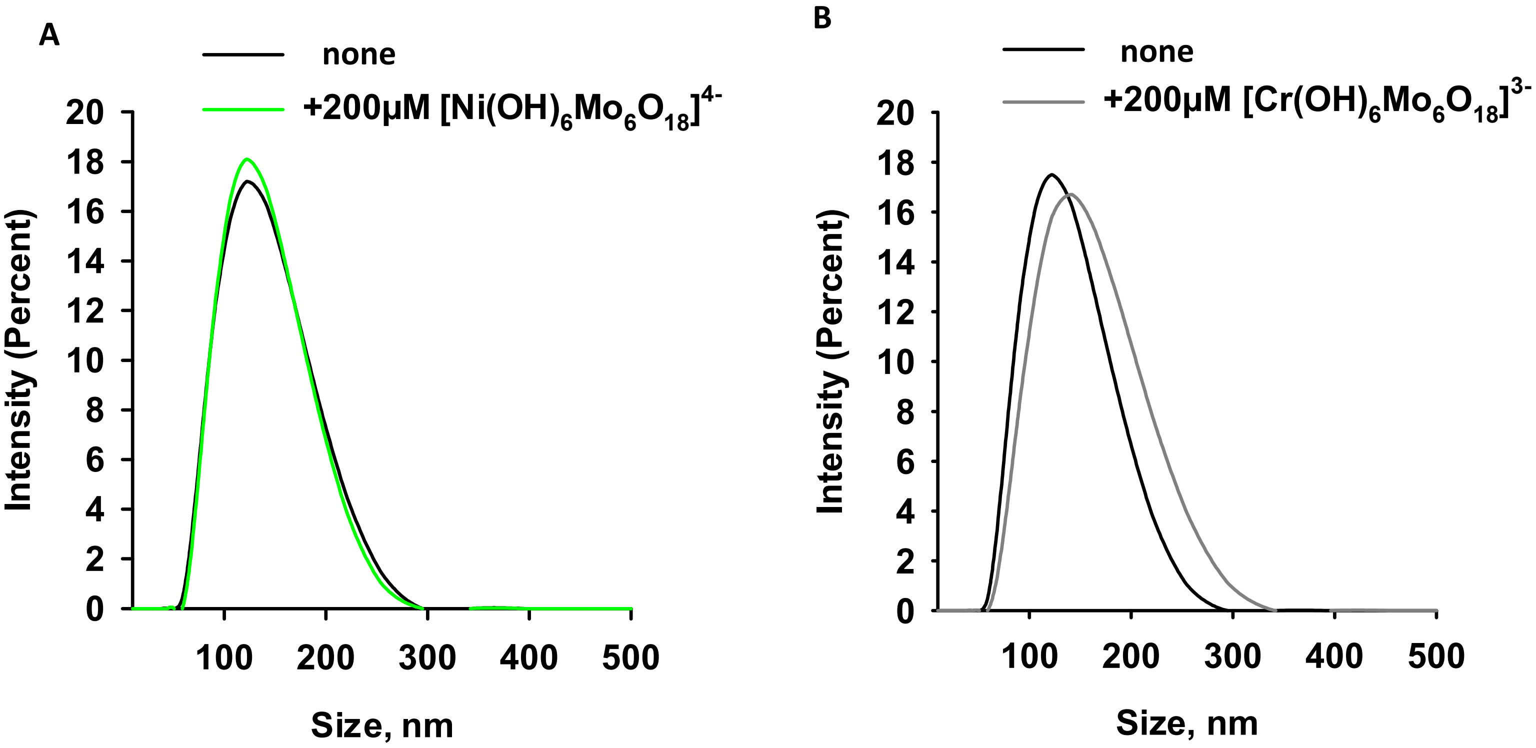

Supplementary Figure 2. Size distribution of liposomes with and without 200 μM POMos and without. The liposomes were prepared from DOPC:CL (90:10%). The lipid concentration was 0.2 mg/ml. The buffer solution consisted of 20 mM Na_2SO_4, 10 mM MES, 10 mM Tris at pH=7.34 and T=25^{0}C.

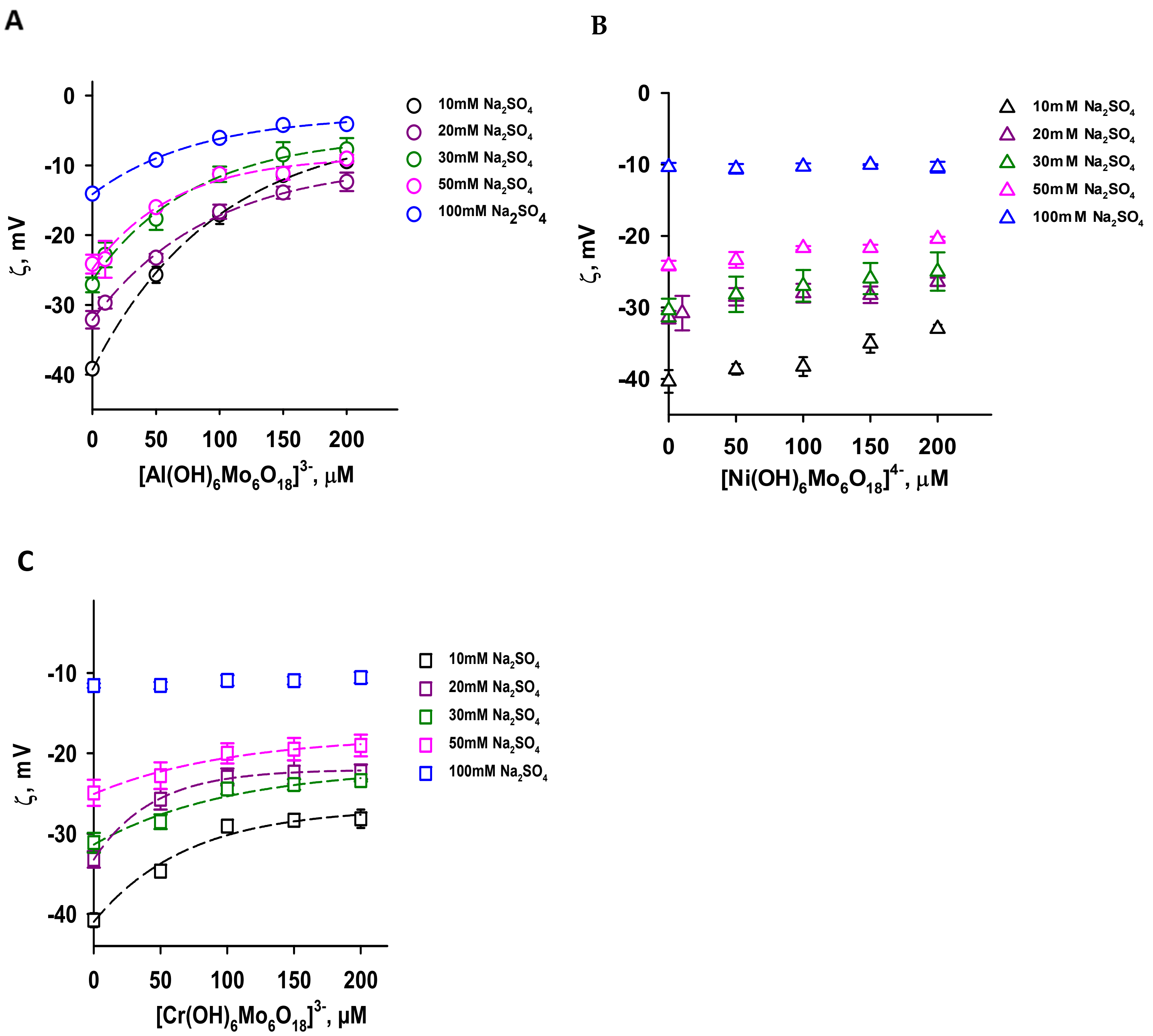

Supplementary Figure 3. Dependence of ζ potential of POMos with different central ions adsorbed on negatively charged liposomes:
(**A**) $[Al(OH)_6Mo_6O_{18}]^{3-}$, (**B**) $[Ni(OH)_6Mo_6O_{18}]^{4-}$ and (**C**) $[Cr(OH)_6Mo_6O_{18}]^{3-}$ at different ionic strength. The liposomes were prepared from DOPC:CL (90:10%) and the lipid concentration was 0.2 mg/ml. The buffer solutions consisted of 10, 20, 30, 50 and 100 mM Na_2SO_4, 10 mM MES, 10 mM Tris at pH=7.34 and T=25 ^{0}C.

Supplementary Table 1. Characterization of the Anderson-Evans-type POMs.

Formula	IR bands in the region 1000-400 cm^{-1}, (cm^{-1})	IR bands according to literature, (cm^{-1})	SXRD lattice constant measured at room temperature, (Å, °)	Reference
$Na_3[Al(OH)_6Mo_6O_{18}]\cdot 8H_2O$	943, 902, 797, 723, 629, 568, 447	947, 845, 650, 574, 530, 447	a = 6.4, b = 10.8, c = 10.9; α = 110, β = 95, γ = 110	Manikumari, Shivaiah et al. 2002
$Na_3[Cr(OH)_6Mo_6O_{18}]\cdot 8H_2O$	943, 910, 804, 611, 572, 504, 414	not reported	a = 10.9, b = 10.9, c = 6.5, α = 108, β = 83, γ = 114	Perloff 1970
$Na_4[Ni(OH)_6Mo_6O_{18}]\cdot 16H_2O$	929, 889, 790, 684, 605, 522, 449	931, 882, 824, 706, 635, 583, 467	a = 8.1, b = 10.6, c = 12.3, α = 70, β = 72, γ = 83	Gumerova, Melnik et al. 2015
$Na_4[Ni(OH)_6W_6O_{18}]\cdot 16H_2O$	958, 893, 642, 580, 484	958, 863, 659, 585, 499	a = 8.1, b = 10.7, c = 12.3, α = 71, β = 72, γ = 85	Rozantsev, Radio et al. 2009
$K_5[H_2SbMo_6O_{24}]\cdot 6H_2O$	925, 877, 650, 551, 430	927, 878, 650, 533, 426	a = 21, b = 10.5, c = 15.3, α = 90, β = 113, γ = 90	Ogawa, Yamato et al. 1988
$K_5[H_2SbW_6O_{24}]\cdot 6H_2O$	948, 885, 690, 601, 559, 449	927, 850, 703, 632, 563, 420	a = 13.8, b = 13.7, c = 12.9, α = 90, β = 90, γ = 120	Naruke and Yamase 1992
$Na_6[TeMo_6O_{24}]\cdot 22H_2O$	952, 908, 885, 653, 605, 534, 441	not reported	a = 10.4, b = 10.7, c = 11.1, α = 90, β = 116, γ = 105	Robl and Frost 1993
$Na_6[TeW_6O_{24}]\cdot 22H_2O$	948, 887, 671, 624, 445	not reported	a = 10.3, b = 10.7, c = 11.2, α = 91, β = 115, γ = 106	Schmidt, Schrobilgen et al. 1986
$Na_2K_6[MnW_6O_{24}]\cdot 12H_2O$	898, 852, 731, 677, 576, 478, 433	912, 863, 700	a = 13.2, b = 13.2, c = 18.4, α = 90, β = 90, γ = 120	Nolan, Burns et al. 2000

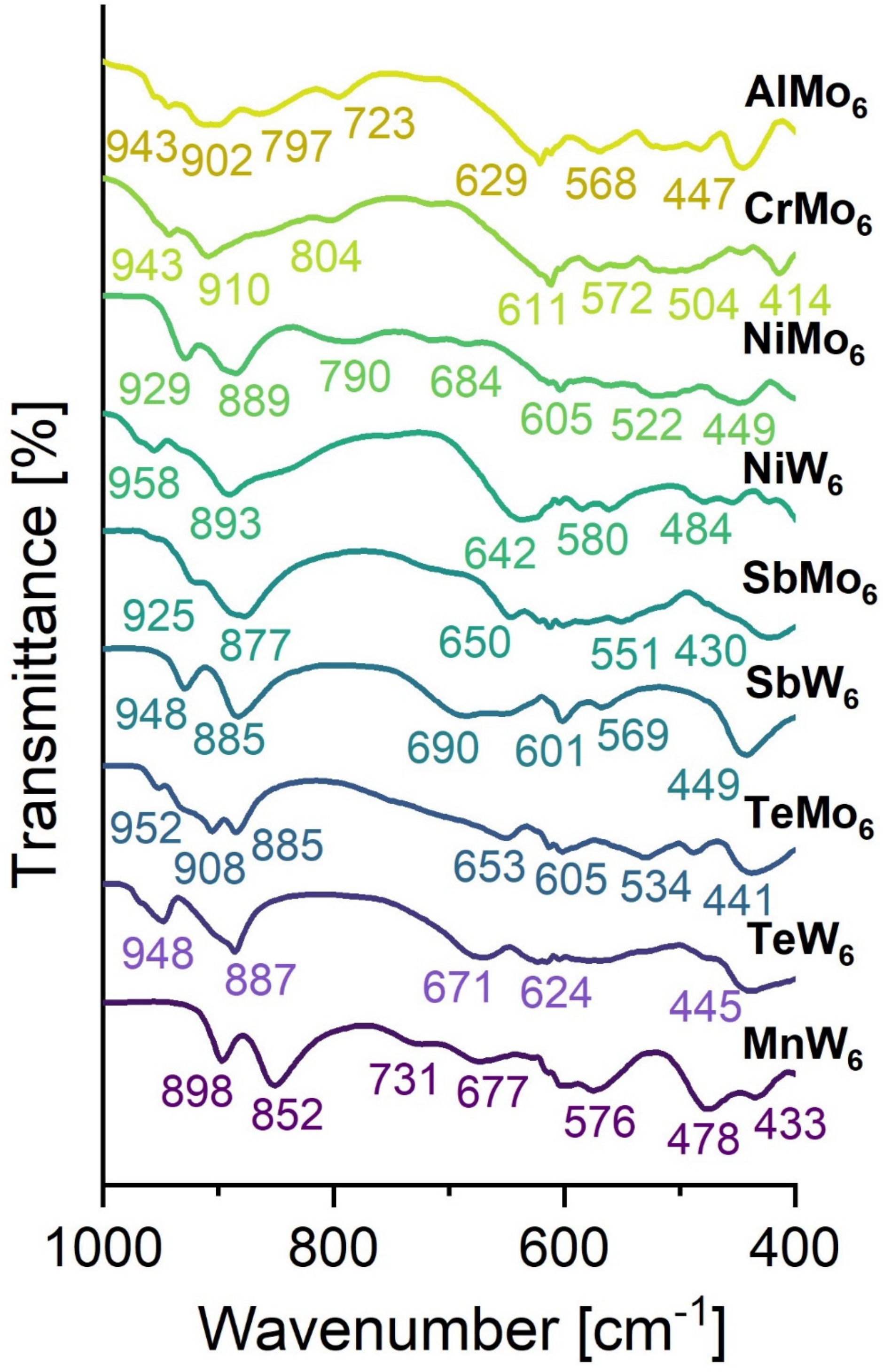

Supplementary Figure 4. IR spectra of Anderson POMs in the fingerprint region 1000 – 400 cm^{-1}.

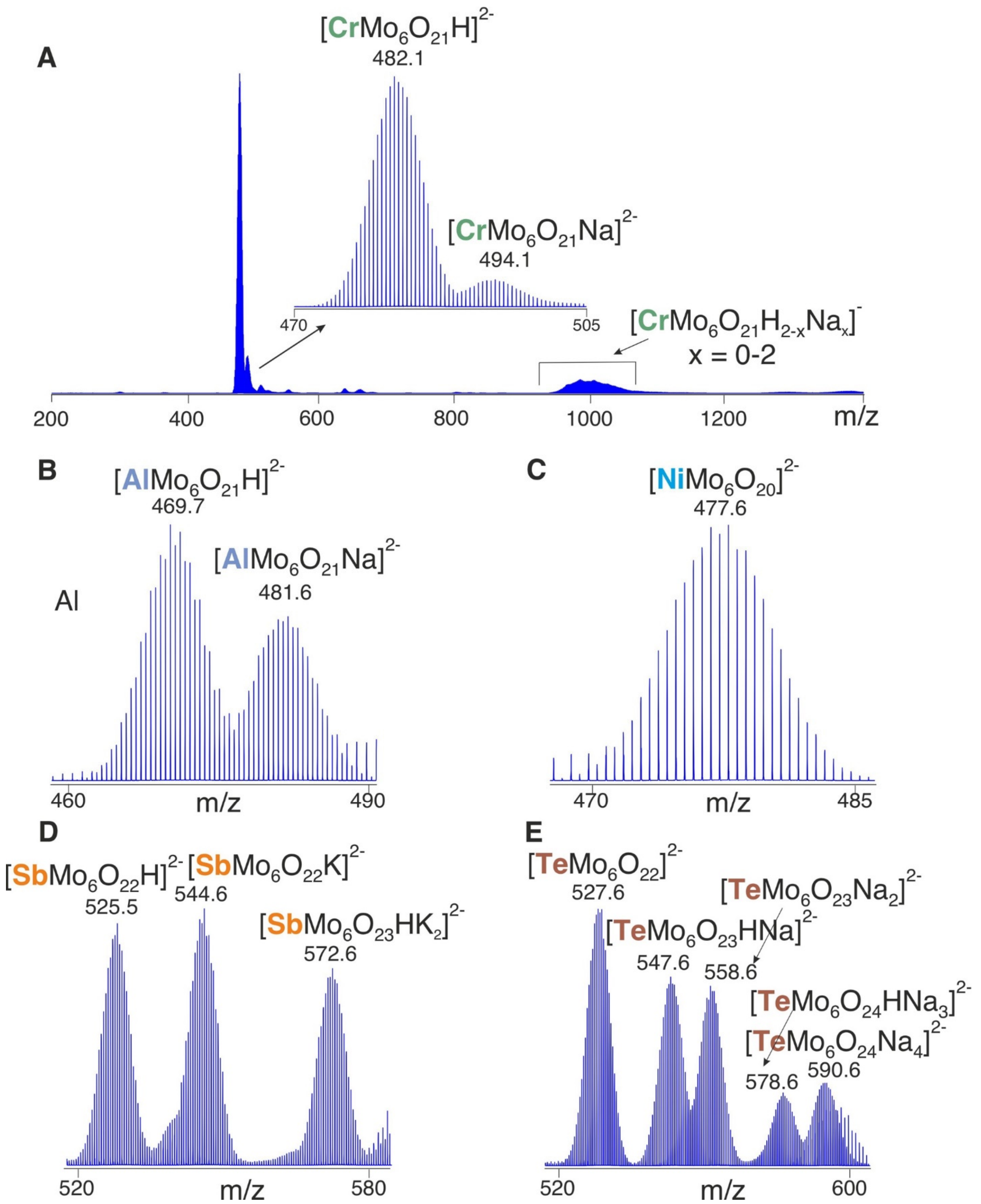

Supplementary Figure 5. ESI mass spectra of 100 μM aqueous solutions of A) $Na_3[Cr(OH)_6Mo_6O_{18}]\cdot 8H_2O$; B) $Na_3[Al(OH)_6Mo_6O_{18}]\cdot 8H_2O$; C) $Na_4[Ni(OH)_6Mo_6O_{18}]\cdot 16H_2O$; D) $K_5[H_2SbMo_6O_{24}]\cdot 7H_2O$; C) $Na_6[TeMo_6O_{24}]\cdot 22H_2O$. The spectra were recorded in negative ion mode within the m/z range of 100 to 1500, and the spectrometer was calibrated using the standard tune-mix to ensure an accuracy of approximately 5 ppm in the m/z region of 100–1500. For $Na_3[Cr(OH)_6Mo_6O_{18}]\cdot 8H_2O$ the whole range is shown (A), for four other POMos just the region where the envelope for XMo_6 is visible.

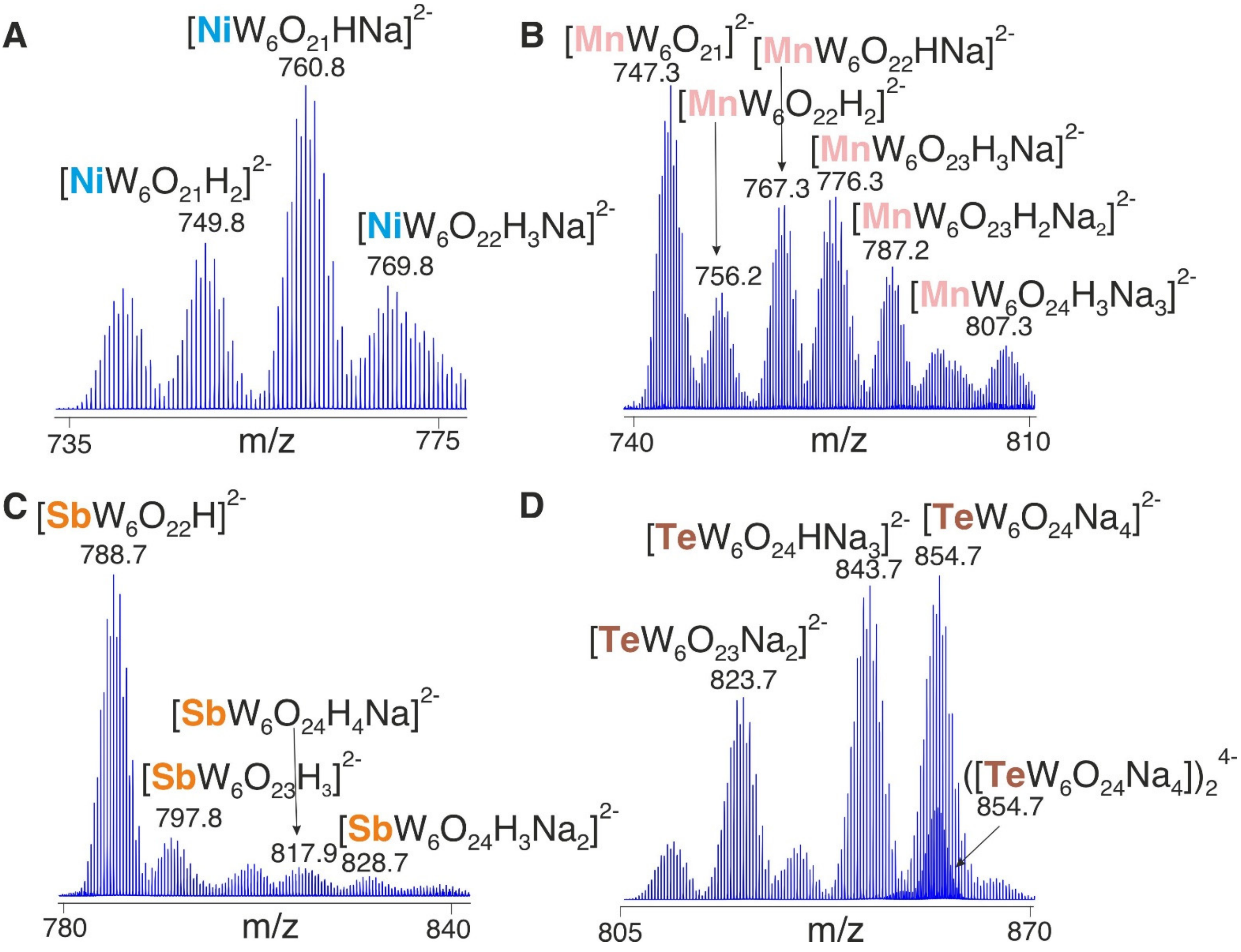

Supplementary Figure 6. ESI mass spectra of 100 µM aqueous solutions of A) $Na_4[Ni(OH)_6W_6O_{18}]\cdot 16H_2O$; B) $Na_2K_6[MnW_6O_{24}]\cdot 12H_2O$ B) $K_5[H_2SbW_6O_{24}]\cdot 6H_2O$; C) $Na_6[TeW_6O_{24}]\cdot 22H_2O$. The spectra were recorded in negative ion mode within the m/z range of 100 to 1500, and the spectrometer was calibrated using the standard tune-mix to ensure an accuracy of approximately 5 ppm in the m/z region of 100–1500. For all POTs, the regions displaying the envelope for the XW_6 species are shown.

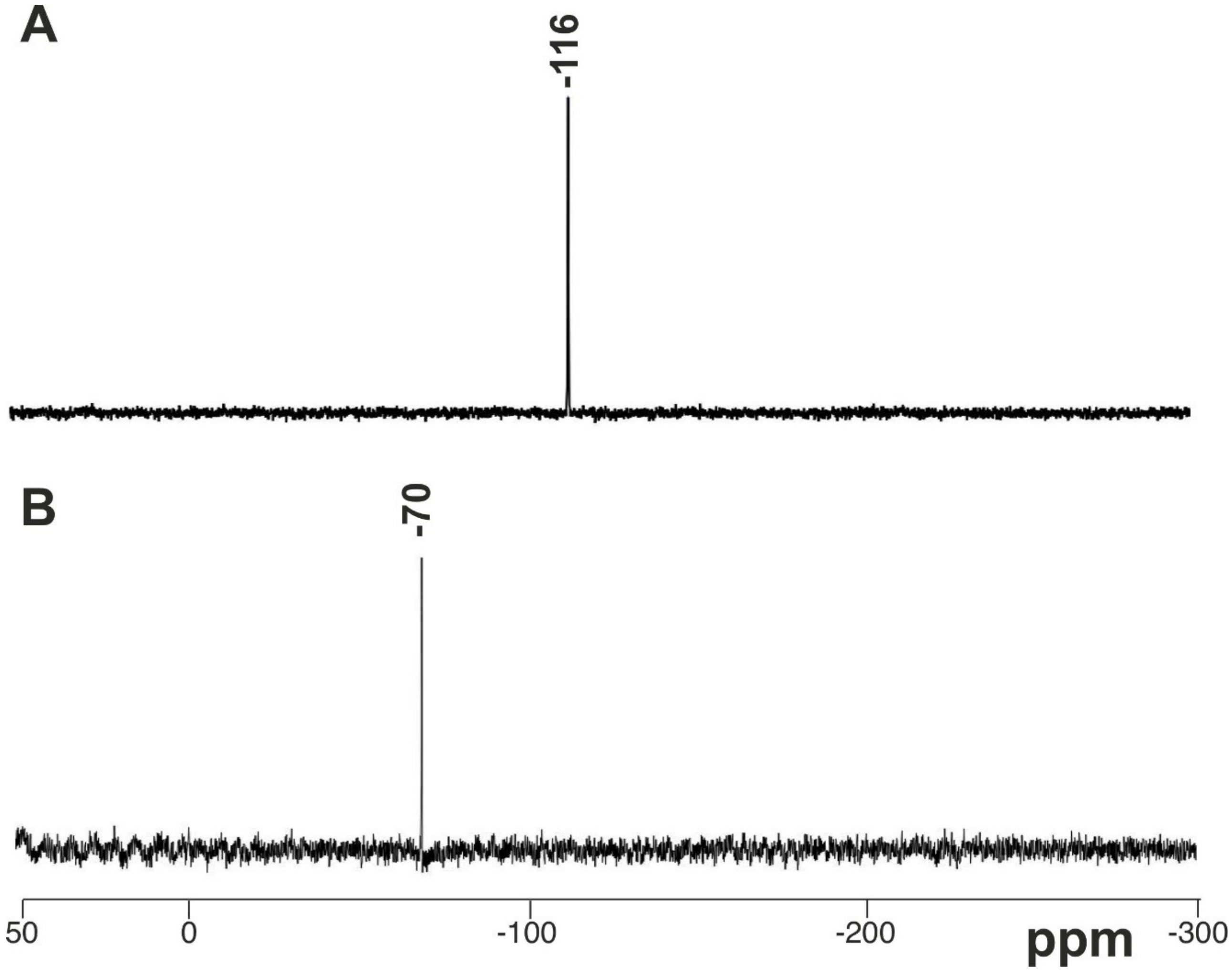

Supplementary Figure 7. ^{183}W NMR spectra of 10 mM solutions of A) $K_5[H_2SbW_6O_{24}]\cdot 6H_2O$ and B) $Na_6[TeW_6O_{24}]\cdot 22H_2O$ in 20 mM Na_2SO_4, 10 mM Tris, 10 mM MES, pH 7.34. The one signal at -70 ppm for SbW_6 and -116 ppm for TeW_6 corresponds to one type of W coordination in the Anderson structure and is in accordance with the literature (Chen, Gang et al., 2004).
